# Practical Hints on the Teeth

**Published:** 1853-07

**Authors:** 


### PRACTICAL HINTS ON THE TEETH.

This is a neat little volume, by W. E Ide, M. D., D. D. S., of Columbus, O. This is a popular treatise for general distribution, and among other things the Dr. treats of first dentition—second dentition, and its regulation—effect of diseased teeth on the general health. Tooth-ache, caries of the teeth, predisposing causes of D ntal caries. The effect of calomel, Tobacco, &c. &c.

The operation of plugging. The use of dentrifices, filing, artificial teeth, &c. These subjects are presented in a plain and practical manner, so that not only the general reader, parents, youth, &c., but the Professional man may be both interested and instructed. It makes a very neat little volume, suitable for the centre table in parlor or office. We thought we had given a notice of this book when first issued, but we find it was not the case.

